# Development of an undergraduate certificate in clinical and translational science: improving competence of the clinical research workforce

**DOI:** 10.3389/fphar.2023.1294534

**Published:** 2023-12-06

**Authors:** Jacqueline Knapke, Michelle Marcum, Angela Mendell, Patrick Ryan

**Affiliations:** ^1^ Department of Family and Community Medicine, University of Cincinnati, Cincinnati, OH, United States; ^2^ Center for Clinical and Translational Science and Training, University of Cincinnati, Cincinnati, OH, United States; ^3^ Cancer Center, University of Cincinnati, Cincinnati, OH, United States; ^4^ Department of Pediatrics, Division of Biostatistics and Epidemiology, Cincinnati Children’s Hospital Medical Center/University of Cincinnati, Cincinnati, OH, United States

**Keywords:** workforce development, clinical and translational science, clinical and translational research, curriculum development, undergraduate research training, clinical research professional

## Abstract

**Introduction:** Academic research centers often struggle to recruit and retain a well-trained and diverse clinical and translational science (CTS) workforce. In particular, the clinical research professional (CRP) career pathway is not well known to undergraduate students and other individuals outside of academic medicine despite being a potential career route. To address these workforce challenges, the CRP Task Force at the University of Cincinnati (UC) aims to train a competent and diverse CRP workforce through targeted educational programming in the UC undergraduate population.

**Methods:** Using a six-step curriculum development process that included: 1) performing a needs assessment, 2) determining content, 3) writing goals and objectives, 4) selecting the educational strategies, 5) implementing the curriculum, and 6) evaluating the curriculum, we designed an undergraduate certificate program in CTS.

**Results:** The needs assessment included both internal and external data gathering to inform curriculum development and program decisions. Content was determined using the Core Competency Framework for the Clinical Research Professional Version 3.1., and program learning outcomes were written with both the competency framework and local workforce needs in mind. Educational strategies were selected based on optimization of available resources and local expertise with an emphasis on interactive didactics complemented by experiential learning. Implementation is underway and evaluation will follow once students begin enrolling.

**Discussion:** By educating an undergraduate student population about CTS methods and career opportunities, we anticipate increased numbers of well-qualified, diverse applicants who pursue CRP careers locally and regionally.

## 1 Introduction

Academic research centers frequently face challenges in the recruitment and retention of well-trained, diverse clinical research professionals (CRPs) for multiple reasons, including a lack of professional identity characterized by insufficient training programs, ill-defined pathways for career advancement, and feelings of low value and burnout (Knapke and Jenkerson, 2022; Knapke and Snyder, 2022; Freel et al., 2023). Freel, et al. (2023) provides a compelling summary of the alarming scale of the problem nationally, and the risk the problem poses to the integrity, quality, and innovation of clinical and translational science (CTS) in the United States. Although CRP retention has not been well-studied in academic medical centers, the turnover rate in healthcare averaged 22.7% in 2022 (‘2023 NSI National Health Care Retention & RN Staffing Report’, 2023). In clinical research organizations (CROs), the average turnover rate from 2017 to 21 was 26.2% (‘2022/23 Clinical Research Organization Insights Report: Managing Talent and Pay in a Competitive Market and Volatile Economy’, 2023). However, it is difficult to compare staffing trends in healthcare and industry to academic research environments. Duke University reported a reduction in CRP turnover from 23% to 16% following implementing a competency-based workforce initiative (Stroo et al., 2020). The COVID-19 pandemic exacerbated CRP workforce problems; one study found that 37% of academic research centers reported decreased staffing and increased turnover as a result of the pandemic (Samuels et al., 2023). Managers and principal investigators (PIs) at the Cincinnati Academic Health Center (AHC) face similar problems to those seen at the national level. The Cincinnati AHC is comprised of three hospitals and one academic institution: the University of Cincinnati Medical Center (UCMC), Cincinnati Children’s Hospital Medical Center (CCHMC), the Cincinnati VA, and the University of Cincinnati (UC), which includes the Colleges of Medicine, Nursing, Allied Health, and Pharmacy on its health sciences campus. Combined, UC and CCHMC employ approximately 1,200 CRPs, but both organizations struggle to recruit and retain a CTS workforce locally, mirroring similar challenges at the national level. During the 2022 fiscal year, turnover rates at both institutions ranged from 18.7% to 37.5%, with the highest turnover rates occurring in the early-to mid-level titles. Turnover rates at these levels introduce a critical roadblock to sustaining high-quality clinical and translational research (CTR) implementation and management.

To overcome these workforce challenges, leaders at the Cincinnati AHC organized a CRP Task Force comprised of key stakeholders from the Center for Clinical and Translational Science and Training (CCTST), the UC Cancer Center, the UC Office of Clinical Research, the Department of Environmental and Public Health Sciences, the UC College of Education, Criminal Justice and Human Services, UC Human Resources, and the Department of Pediatrics/CCHMC. The goal of the Task Force is to develop and implement strategies to recruit, train, and retain CRPs to support the clinical research enterprise at the Cincinnati AHC. Three workgroups within the task force were formed to focus on recruitment, education, and retention. The work described in this paper was completed by the education workgroup, whose goals are to support and promote for-credit training opportunities and non-credit professional development for new and existing CRPs.

Despite the critical role CRPs play in the generation of evidence to support better health outcomes for both individuals and populations, this career pathway is not well known to undergraduate students and other individuals outside of academic medicine despite being a potential career route. Summer research programs for undergraduates and medical students are the most common method for introducing students to research (Black et al., 2013; Kolber et al., 2016; Howell et al., 2019; Avila et al., 2022). Summer programs can also be an effective way to introduce underrepresented minority students to research and prepare them for CTS career pathways (Ghee et al., 2016; Smalley and Warren, 2020; Prince et al., 2023). In Arkansas, an undergraduate curriculum rooted in a real-world CTS study was developed, offered, and evaluated, demonstrating high satisfaction among learners (James et al., 2023). Temple University School of Medicine requires medical students to complete 2 week training in CTS and one CTS scholarly activity during their 4 years of medical school (Feldman, 2015). Evidence suggests that virtual programming is an effective training method when in-person is not feasible (Corson et al., 2021; Lemacks et al., 2022; James et al., 2023). Evidence also suggests that research experience during undergraduate study increases students’ awareness of career options, improves their preparation for graduate training, and ultimately impacts their decisions to pursue advanced degrees and careers related to research (Seymour et al., 2004; Hunter et al., 2007; Adedokun et al., 2012; Yaffe et al., 2014).

Locally, we coordinate several efforts to introduce students to research principles and careers. Every semester, the workforce development core of our Center for Clinical and Translational Science and Training visits undergraduate courses across several programs to introduce students to CTS careers and training opportunities. Research 101 is an asynchronous research primer available to medical students and summer research students (Blackard et al., 2022; 2023). Every summer, the Summer Undergraduate Research Fellowship (SURF) program awards research fellowships to 150 undergraduate students from UC and other institutions. The Office of Undergraduate Research on UC’s main campus also provides programs and resources to help students access research experiences across an array of disciplines. However, currently, there is no formal training pathway specific to CTS that results in a major, minor, or certificate for undergraduates at UC. In order to introduce and better prepare undergraduate students for careers in CTS, the education workgroup of the CRP Task Force at the Cincinnati AHC sought to develop a competency-based, for-credit undergraduate certificate program by undertaking a six-step curriculum development process.

## 2 Pedagogical frameworks

Two pedagogical frameworks informed our task: existing CRP competencies and an established six-step curriculum development method. We began with CRP competencies that were developed by a national consortium of medical association leaders and industry collaborators called the Joint Task Force (JTF) for Clinical Trial Competency, organized by the Multi-Regional Clinical Trials (MRCT) Center of Brigham and Women’s Hospital and Harvard. This framework has undergone multiple iterations between 2014-20; we utilized the most recent “Core Competency Framework for the Clinical Research Professional Version 3.1.” (Sonstein et al., 2014; 2020; JTF Task Force, 2017). The Core Competency Framework for the CRP Version 3.1 defines 49 competency statements that address CRP knowledge, skills, and attitudes under 8 scientific domains: 1) Scientific Concepts and Research Design, 2) Ethical and Participant Safety Considerations, 3) Investigational Products Development and Regulation, 4) Clinical Study Operations (Good Clinical Practice), 5) Study and Site Management, 6) Data Management and Informatics, 7) Leadership and Professionalism, and 8) Communications and Teamwork. Additionally, we utilized a six-step curriculum development process described by Schneiderhan, Guetterman and Dobson (2019) that included: 1) performing a needs assessment, 2) determining content, 3) writing goals and objectives, 4) selecting the educational strategies, 5) implementing the curriculum, and 6) evaluating the curriculum.

## 3 Learning environment

UC is designated a “very high research activity” university by the Carnegie Commission, holding ∼$206.6 million in grants in 2019 with $89.4 million coming from the NIH. The College of Medicine received $105.3 million in sponsored awards in 2019 and was ranked in the top 38% of medical schools for research in the 2020 *U.S. News and World Report* rankings. The College of Medicine is composed of 23 departments, 5 basic science and 18 clinical, which retain in excess of 2,000 faculty. CCHMC is a 700-bed non-profit organization serving as the AHC’s major teaching facility for pediatrics and as the only children’s hospital in the Cincinnati metropolitan area (population 2.3 million). Of 184 pediatric institutions surveyed nationally, CCHMC is consistently ranked top 3 in the Honor Roll of America’s Best Children’s Hospitals compiled by *U.S. News & World Report.* CCHMC has a major emphasis on research and held over $240 million in grants with over $161 million coming from the NIH in 2019. The two institutions are located on the same campus and have a long record of close collaboration. There are constant interactions between researchers and clinicians, and CCHMC faculty hold dual appointments in the UC College of Medicine. The institutions are administratively linked in many endeavors ranging from clinical to research and education. This emphasis on research impacts the learning environment in a multitude of ways (e.g., collaborations across faculty research, training grants, core facilities, and CRP training). UC’s undergraduate population includes approximately 40,000 students, with a quarter identifying as a racial or ethnic minority. Reaching a small fraction of those students and introducing them to CTS principles and career opportunities could have a major impact on local workforce development.

## 4 Methods

The education subgroup of the CRP Task Force followed the six-step curriculum development process outlined by Schneiderhan, Guetterman and Dobson (2019) to design an undergraduate certificate program in CTS.

### 4.1 Needs assessment

An educational needs assessment is a data-gathering exercise to understand what the needs for a particular discipline or group of learners are and why a curriculum should be developed and implemented. It can include a wide range of data sources: consultations with those familiar with the field and/or potential learners, data-driven descriptions of an educational gap in a particular discipline, or accreditation or regulatory specifications (e.g., Accreditation Council for Graduate Medical Education requirements) that must be achieved.

Our needs assessment included four components. We gathered information from local CTS leaders about the undergraduate majors where most of their employees come from, and then we conducted an internal review with program directors from these programs, seeking to better understand the major curricula and their students’ educational needs and career interests. We also provided the directors with an overview of CTS careers and noted their perceptions of how well an undergraduate certificate would fit the needs and interests of their student populations. We worked with local CTS leaders and Human Resources to conduct an internal review of employment needs within the local CTS research workforce. We reviewed and summarized relevant competitor programs (both internal and external). When reviewing external programs, we focused on direct competitors: undergraduate certificate programs offered by 4-year universities. Finally, we completed an external market analysis to better understand career opportunities for potential graduates. These components occurred simultaneously over approximately 6 months and are described in Table 1.

**TABLE 1:** Needs assessment components.

Data source	Data type	Component	Brief description
Internal	Qualitative	Review of Undergraduate Major Program Directors	Meetings with program directors from undergraduate majors with anticipated high levels of student interest
Internal	Qualitative & Quantitative	Review of Local CTS Workforce Needs	Meetings with CTS research managers and directors Review of human resource data related to CRP recruitment and retention
Internal & External	Qualitative & Quantitative	Review of Relevant Competitor Programs	Web-based research to identify and summarize existing programs both internally and at external institutions
External	Quantitative	Market Analysis of CTS Career Opportunities	Market analysis using Lightcast, an external labor analytics company

### 4.2 Determine content

The next step was to consider areas of content that should be included in the curriculum, making decisions about what to focus on and prioritize. Subject matter experts are important to include in this step of the process, as they are familiar with large thematic areas as well as content specifics that could inform the organization of the training program or coursework. Our process for determining content included two components: a review and prioritization of existing competencies and a review of relevant undergraduate courses that already existed at our institution.

Using the Core Competency Framework for the CRP Version 3.1 as a foundation, we carefully reviewed the competencies with subject matter experts (including a CRP, a CRP manager, and a CTS director) to determine areas of prioritization that would best support the types of studies commonly conducted at our AHC. We categorized each competency as essential, important, or not needed in an entry-level CRP position. We also adjusted competency language when necessary to make competency achievement feasible in undergraduate learners, e.g., lowering the level of competency to “understanding” or “summarizing” rather than “analyzing” or “evaluating” (Bloom, 1956). We also noted competencies that should be introduced in the certificate curriculum, but that would be further explicated as part of employee onboarding and/or required training.

The second component of our content determination process was reviewing existing courses at our institution that were relevant to the content areas within the JTF competencies. This entailed searching for several keywords within the course catalog, collating a list of potential course numbers, and then examining the internal course management system and reviewing details such as course descriptions and student learning outcomes for alignment with program competencies. The “determine content” step of the curriculum development process resulted in an early draft of required courses for the certificate program.

### 4.3 Write goals and objectives

Once content areas were identified and prioritized using the Core Competency Framework for the CRP Version 3.1, we established goals and objectives for the certificate program. Schneiderhan, Guetterman and Dobson (2019) draw an important distinction between goals and objectives: goals are broad and general statements of knowledge or skill that learners should attain, while objectives are specific and measurable outcomes learners should achieve after program completion. Goals were drawn from the content areas outlined in step two, and objectives were more specific summaries of the competencies to be achieved. We chose the verbs for our objectives carefully, focusing on achievement that could be measured (e.g., describe or compare) as opposed to more vague characterizations of achievement (e.g., know or appreciate).

### 4.4 Select educational methods

Given the plethora of methods available to contemporary educators (e.g., lectures, flipped classrooms, case studies, experiential learning, hands-on skill delivery, web-based synchronous or asynchronous learning, role plays, etc.), selecting strategies to effectively facilitate the curriculum was a critical step in the development process. Strategy selection required us to give holistic consideration to several elements of the program that had been identified and described in the first three steps: the needs of the disciplinary field and the learners (step 1), effective content delivery based on these needs (step 2), and the optimal methods to support and measure achievement of program learning objectives (step 3). As we considered strategies to teach undergraduate students introductory principles of CTS, we consulted with stakeholders who had knowledge regarding two key facets that would impact our training strategies: 1) the real-life challenges and opportunities when engaging with an undergraduate student population, and 2) the on-the-job needs of a new CRP hire. We also consulted with potential instructors of the content areas to talk through benefits and barriers of different methods of content delivery.

### 4.5 Curriculum implementation

Implementation of the curriculum encompassed several discrete but related tasks such as identifying the necessary resources (e.g., personnel, time, facilities, and budget), obtaining any necessary internal and external stakeholder support, designing an educational management plan that includes logistical details regarding curriculum components, educational methods, barrier mitigation, and other implementation processes such as learner recruitment, retention, and program completion. The final step is actual delivery of the curriculum to learners, sometimes via pilot rollouts or a phased approach over time. In our case, several of these tasks had been discussed in earlier steps of the development process but this step brought them all together in a formal program proposal required by our institution for the new program approval process. During this step, we brainstormed and contacted potential course instructors, discussed admission requirements, considered budgetary or logistical limitations, and identified existing resources at our institution that could be leveraged to implement or improve educational methods.

### 4.6 Curriculum evaluation and improvement

Evaluation is an essential element to any educational program, not only for continuous program improvement but also for reporting program outcomes to key stakeholders. Evaluation is iterative in nature, often including formative (process) and summative (outcome) evaluation methods. Schneiderhan, Guetterman and Dobson (2019) describe a five-step curriculum evaluation process: 1) determine how evaluation results will be used, 2) identify the best metrics for evaluating objective achievement, 3) collect data, 4) analyze data, and 5) improve the program using results. Our evaluation planning included an exploration of data that would be obtained through the common administrative processes at our institution, identified gaps within those data that we determined were important in order to measure program success, and developed measures and accompanying processes to collect data in order to effectively evaluate the program. We gave careful consideration to whom these data would be important to (e.g., key stakeholders, institutional administrators, and potentially granting agencies who may support our training efforts), and we discussed ways to embed evaluation measures into learner assessments at the course level. We also developed a utilization plan for evaluation results, designed to promote regular review of evaluation data and integration of curriculum changes annually, as needed and informed by evaluation results.

## 5 Results

### 5.1 Needs assessment

#### 5.1.1 Review of undergraduate major program directors

Our information-gathering from local CTS leaders identified Biological Sciences, Medical Sciences, Psychology, and Public Health as the most common undergraduate majors held by their employees, allowing us to select these as programs that could potentially yield high numbers of interested students. Meetings with the program directors of these four majors led us to conclude that an undergraduate certificate was the appropriate educational path to pursue due to limited room for additional credits in student major curricula, as well as student preference for a certificate rather than a minor. Program directors were unanimously in favor of development of the program. The majority were unaware of CTS career pathways that might be appropriate for their graduates, but felt there would be high levels of interest from their students and that the skills taught in such a certificate program would be new yet complementary to content offered through existing courses. These programs also offer significant numbers of majors: approximately 900 in Biological Sciences, 375 in Medical Sciences, 1,000 in Psychology, and 125 in Public Health.

#### 5.1.2 Review of local CTS workforce needs

In addition, our meetings with CTS research managers and directors at the Cincinnati AHC led to a better understanding of the workforce challenges they have faced, revealing that although recruitment and retention have long plagued the CTS field, the COVID-19 pandemic exacerbated the problem, and the workforce has not recovered. The CRP workforce at the Cincinnati AHC includes approximately 1,200 positions, encompassing titles from clinical research assistant (high school diploma required) to clinical research director (master’s required, some staff have doctorates). UC employs approximately 400 CRPs and CCHMC employs approximately 800 CRPs. Additionally, several corporate Clinical Research Organizations (CROs) operate large offices in the Cincinnati area, primarily specializing in clinical trials. In 2022, turnover rates at the Cincinnati AHC varied from 20% to 37.5% at CCHMC and 19%–32% at UC, with the highest rates occurring in the entry- and median-level position titles. A search of job postings in July 2023 using “clinical research” as a keyword results in 389 results at UC and 247 at CCHMC.

#### 5.1.3 Review of relevant competitor programs

We reviewed potential competitor programs both at UC and at external institutions. We found no similar programs locally, but web-based research allowed us to summarize primary components of external competitor programs as shown in Table 2. An important discovery within this review was that all of the existing programs in our geographic region were only open to students enrolled at those universities (i.e., UC students could not enroll in them).

**TABLE 2:** External undergraduate certificate programs with institution, program name, mode of offering, number of credits, and required courses.

Institution, *Program Name,* mode of offering	Number of credits or courses	Required courses
The Ohio State University	13 credits	- Medical Terminology for the Health
*Certificate in Clinical Trials Sciences*		Professions
Online—both synchronous and asynchronous		- Drug Discovery, Development and Delivery
		- Clinical Trials from Concept to Launch
		- Clinical Trials Data Management and Monitoring
Temple University	18 credits	- Health and Disease in American Society
*Certificate in Health Research*		- Statistical Methods in Sociology
Hybrid		- Research Design and Methods
		- Two additional course electives from a list
University of California—Berkeley	12 credits	- Introduction to Clinical Research: Clinical Trial
		Phases and Design
*Certificate in Clinical Research Conduct and Management*		
Hybrid		- Clinical Trial Planning: Protocol Development
		Data Management and Clinical Site Activities
		- Clinical Trial Implementation: Site Initiation
		Subject Recruitment, Monitoring and Safety
		Reporting
		- Clinical Trial: Data Analysis, Regulatory Audits
		Vendor Selection and Project Management
University of Hawaii at Manoa	6 courses	- Introduction to Cancer
*Clinical Research Professional Certificate Program*		- Introduction to Clinical Research
Online		- Human Subjects Protection
		- Community-Based Participatory Research
		- Clinical Research Advanced Topics
		- Clinical Trials Ethics
University of Kentucky	12–15 credits	- Research in Human Health Sciences
*Certificate in Research in Human Health Sciences*		- Research Experience in Health Sciences
Hybrid		- Out-of-Discipline Course at 300 level or higher
		- Dissemination Requirement/Presentation-Manuscript Preparation
Washington University in St. Louis	21 credits	- Fundamentals of Clinical Research
*Certificate in Clinical Research Management*		Management I
Hybrid		- Fundamentals of Clinical Research
		Management II
		- Pharmacology for Clinical Research
		- Research Ethics and Regulatory Affairs
		- The Business of Clinical Research
		- Introduction to Data and Information
		Management in Health Sciences
		- Practicum/Capstone

#### 5.1.4 Market analysis of CTS career opportunities

In addition to the local market needs described above, we worked with UC Online to conduct a market analysis using Lightcast. (Lightcast—Labor Market Analytics, 2023). The Lightcast report, run in June 2023, found over 13,000 unique job postings for CRP positions with a median advertised salary of $78,600. The median income for CRPs from June 2022 to May 2023 showed a steep increase of 16% because the number of open jobs currently exceeds the supply of qualified applicants. The minimum education levels for employment were: 11% required a high school diploma or GED, 11% required an associate’s degree, and 78% required a bachelor’s degree. Given that large majority of entry level jobs require a bachelor’s degree, we determined that students graduating with a baccalaureate degree would benefit from targeted training in CTS in the form of a certificate that would complement their major curricula. Colleges and universities were the top employers of CRPs.

### 5.2 Determining content

The competency review process generated a final list of prioritized competencies that are essential and/or important to an undergraduate learner who might begin an entry-level position after graduation. The final list acknowledged that while all of the competencies are essential or important to a CRP over the lifetime of their career, many of them will be acquired with onboarding, professional development training, and career experience; thus, some competencies need only to be introduced to undergraduate trainees so that they are aware of common processes, terms, or aspects of CTS. Table 3 provides details on how competencies were prioritized for undergraduate education. Changes to competency language in order to bring them to an undergraduate/introductory level are provided in red. Competencies that may be supported by completion of the Collaborative Institutional Training Initiative Program (CITI) training as part of employment onboarding are noted with an asterisk. Alignment with competencies was the major driving force of the content determination process, but careful review of the external programs summarized in Table 2 was also informative as we considered different ways to organize the topics into required courses.

**TABLE 3:** Core competency framework for the CRP version 3.1, prioritized for undergraduate education.

Scientific domain/Core competency	Essential	Important	Not needed
1. Scientific concepts and research design: Encompasses knowledge of scientific concepts related to the design and analysis of clinical trials			
1.1 Apply principles of biomedical science to investigational product discovery and development and health-related behavioral interventions	x		
1.2 Identify scientific questions that are potentially testable clinical research hypotheses		x	
1.3 Identify the elements and explain the principles and processes of designing a clinical study	x		
1.4 Maintain awareness of new technologies, methodologies and techniques which enhance the conduct, safety and validity of the clinical study			x
1.5 Critically analyze clinical study results			x
**2. Ethical and Participant Safety Considerations: Encompasses care of patients, aspects of human subject protection, and safety in the conduct of a clinical trial**			
2.1 Differentiate between Describe standard of care and clinical study activities	x		
2.2 Define the concepts of “clinical equipoise” and “therapeutic misconception” as they relate to the conduct of a clinical study		x	
2.3 Apply relevant national and international principles of human subject protections and privacy throughout all stages of a clinical study*		x	
2.4 Explain the evolution of the requirement for informed consent from research participants and the principles and content of the key documents that ensure the protection of human participants in clinical research*		x	
2.5 Describe the ethical issues involved when dealing with vulnerable populations and what additional safeguards should be in place for those populations*	x		
2.6 Evaluate and apply an understanding of the relevant ethical issues and cultural variation as it applies to the commercial aspects of the clinical research and investigational product development process			x
2.7 Explain why inclusion, exclusion, and other criteria are included in a clinical protocol to assure human subject protection	x		
2.8 Summarize the principles and methods of distributing and balancing risk and benefit; through selection and management of clinical study subjects*	x		
**3. Investigational Products Development and Regulation: Encompasses knowledge of how investigational products are developed and regulated**			
3.1 Discuss the historical events that precipitated the development of governmental regulatory processes for investigational products*	x		
3.2 Describe Summarize the roles and responsibilities of the various institutions participating in the investigational products development process		x	
3.3 Explain Summarize the investigational products development process and the activities which integrate commercial realities into the life cycle management of medical products		x	
3.4 Summarize the legislative and regulatory framework that supports the development and registration of investigational products and ensures their safety, efficacy and quality	x		
3.5 Describe Summarize the specific processes and phases that must be followed for the regulatory authority to approve the marketing authorization for a medical product	x		
3.6 Describe the pre- and post- approval safety reporting requirements of regulatory agencies		x	
3.7 Appraise the issues generated and the effects of global expansion on the approval and regulation of medical products			x
**4. Clinical Study Operations (Good Clinical Practice): Encompasses study management and GCP compliance; safety management (adverse event identification and reporting, post-market surveillance, and pharmacovigilance), and handling of investigational product**			
4.1 Explain Summarize how the design, purpose, and conduct of individual clinical studies fit into the goal of developing a new intervention		x	
4.2 Describe the roles and responsibilities of the clinical investigation team as defined by Good Clinical Practice Guideline	x		
4.3 Evaluate Understand the design, conduct and documentation of clinical studies as required for compliance with Good Clinical Practice Guideline		x	
4.4 Compare and contrast Summarize the regulations and guidelines of global regulatory bodies relating to the conduct of clinical studies		x	
4.5 Describe Summarize appropriate control, storage and dispensing of investigational product		x	
4.6 Differentiate Summarize the types of adverse events (AEs) that may occur during clinical studies and explain the identification process and reporting requirement to IRBs/IECs, sponsors and regulatory authorities	x		
4.7 Describe how global regulations and guidelines assure human subject protection and privacy during the conduct of clinical studies*		x	
4.8 Describe the role and process of monitoring a clinical study	x		
4.9 Describe the role and purpose of clinical study audits	x		
4.10 Describe the various methods by which safety issues are identified and managed in clinical studies	x		
**5. Study and Site Management: Encompasses content required at the site level to run a study (financial and personnel aspects). Includes site and study operations (not encompassing regulatory/GCPs)**			
5.1 Describe the methods used to determine whether to sponsor, supervise or participate in a clinical study			x
5.2 Develop and manage Understand the functional and operational efficiencies and personnel resources necessary to conduct a clinical study		x	
5.3 Describe the management and training approaches to mitigate risk to improve clinical study conduct	x		
5.4 Develop strategies to manage participant recruitment, retention, compliance and track study activities	x		
5.5 Identify the legal responsibilities, liabilities and accountabilities that are involved in the conduct of clinical studies		x	
5.6 Identify and explain the specific procedural, documentation and oversight requirements of principal investigators, sponsors, CROs and regulatory authorities that relate to the conduct of a clinical study as they relate to the CRP	x		
5.7 Identify, organize, analyze and report Describe why project performance evaluation is necessary for comprehensive management of a clinical study	x		
**6. Data Management and Informatics: Encompasses how data are acquired and managed during a clinical trial, including source data, data entry, queries, quality control, and correction and the concept of a locked database**			
6.1 Describe the role and importance of statistics and informatics in clinical studies		x	
6.2 Describe the origin, flow, and management of data through a clinical study		x	
6.3 Describe best practices and resources required for standardizing data collection, capture, management, analysis, and reporting	x		
6.4 Describe, develop, and implement processes for data quality assurance	x		
**7. Leadership and Professionalism: Encompasses the principles and practice of leadership and professionalism in clinical research**			
7.1 Describe and apply the principles and practices of leadership, management and mentorship in clinical research			x
7.2 Identify ethical and professional conflicts associated with the conduct of clinical studies and implement procedures for their prevention or management*	x		
7.3 Identify and apply the professional guidelines and codes of ethics that apply to the conduct of clinical research*	x		
7.4 Describe the impact of regional diversity and demonstrate cultural competency in clinical study design and conduct	x		
**8. Communications and Teamwork: Encompasses all elements of communication within the site and between the site and sponsor, CRO, and regulators. Understanding of teamwork skills necessary for conducting a clinical trial**			
8.1 Describe the importance of team science and methods necessary to work effectively with cross-functional, multidisciplinary and inter-professional research teams, which may include external partners	x		
8.2 Discuss the relationship and appropriate communication between Sponsor, CRO and clinical research site	x		
8.3 Effectively communicate the content and relevance of clinical research findings to colleagues, advocacy groups and the non-scientist community		x	
8.4 Describe the components of a traditional scientific publication		x	

The bold values are the domain names while the sub-numbers (1.1, 1.2 etc) are the competencies under those domains. Domain 1 is a different font than the other domains.

The second piece of the determination and prioritization of content entailed a thorough review of existing courses at UC that might be incorporated into a certificate curriculum. No courses were identified that were specific enough in scope to be a good fit for the certificate. Completion of this step of the curriculum development process led to development of a 12-credit curriculum detailed in Table 4, including general topics for each course as well as competency domains.

**TABLE 4:** Proposed curriculum for undergraduate certificate in CTS, with JTF competency domains.

Course name (credits) mode of offering	Topics	Competency domains
**Healthcare Exploration Through Patient Care** (3) In-person	• Core competencies for healthcare professionals, outlined by the American Association of Medical Colleges (*The Core Competencies for Entering Medical Students*, 2023)	1, 2, 7, 8
	• Internship experience in hospital setting	
**Introduction to Clinical and Translational Science** (3) Online	• CTR spectrum	1, 2, 6
	• Career tracks	
	• Ethics & Human Research Participant Regulations	
	• Grants vs. Industry-Sponsored	
	• Study methods	
	• Data collection/management	
	• Introductory biostatistics/informatics	
	• Diversity, Equity, and Inclusion (DEI) & health disparities	
**Fundamentals of Clinical Trials** (3) Online	• GCP	2, 3, 4, 5
	• Safety and Adverse Events	
	• Regulatory	
	• Phases of clinical trials	
	• Vested stakeholders	
**Healthcare Exploration through Clinical and Translational Research** (3) Hybrid	• Leadership	5, 7, 8
	• Professionalism	
	• Communication	
	• Team Science	
	• Project management (participant recruitment and retention, timelines, budgets, workflows, team roles)	
	• Internship experience with a CTS research office	

The bold values are the names of the courses.

### 5.3 Writing goals and objectives

Steps two to three were completed concurrently: as the curriculum was being discussed, so were the goals and objectives (or student learning outcomes, in the language of our specific institution) being developed. The overarching goal for the certificate program is broad and general, following the guidelines of Schneiderhan, Guetterman and Dobson (2019): to introduce undergraduate students to the principles of CTS in order to prepare them for CTS research careers upon graduation. The objectives for the certificate program are much more specific, measurable, and grounded in the Core Competency Framework for the CRP Version 3.1:

• Explain Good Clinical Practice according to the NIH.
• Summarize the fundamental processes of clinical and translational science, including participant recruitment, addressing diversity, equity, inclusion, and accessibility, data collection and management, study site management, and financial management to support clinical research activities.
• Describe the stages of clinical trials and their relevant regulatory components.
• Demonstrate project management and communication skills in a team-based research setting.
• Connect the goals and outcomes of clinical and translational research to the goals and outcomes of patient care and population health.

### 5.4 Selecting educational methods

Training undergraduate students was a new concept to the majority of CRP Task Force members, so we relied heavily on the expertise of our undergraduate program directors to understand student needs when considering how best to deliver the curriculum. Two key points guided decision-making of certificate educational methods: undergraduate students are often limited in time and the number of credits they can devote to non-major curriculum requirements, and although the AHC is less than 1 mile from UC’s main campus, most undergraduates are not physically present on the AHC campus. Requiring students to attend numerous in-person courses on the AHC campus would likely prohibit their participation in the program.

Balancing student needs with the desired level of competency upon program completion, we determined a hybrid program was optimal. Two experiential learning courses were incorporated into the curriculum: a paid work experience course where students attend a weekly, 2.5 h evening seminar and work as a patient care team member at the UCMC approximately 12 h per week over one semester, and a second research experience where students work in a clinical and translational research unit at UC or CCHMC over the course of a semester while also participating in an online weekly seminar. To balance these in-person requirements with more flexibly scheduled courses, the remaining two required courses will be offered online.

Two national professional societies offer CRP credentialing: the Society of Clinical Research Associates (SOCRA) and the Association of Clinical Research Professionals (ACRP). Although the certificate will not be credentialed through SOCRA or ACRP, we plan to explore that possibility post-implementation. Both SOCRA and ACRP accept some form of CRP-related coursework as part of their credentialing process (ACRP, 2023; SOCRA, 2023). Students will be provided with certification information as part of the program as well, and they may choose to pursue CRP certification after graduation. The undergraduate certificate curriculum should help students move toward certification if desired and allow them to choose the credentialing society that best meets their needs.

### 5.5 Curriculum implementation and curriculum evaluation/improvement

We are currently in steps five to six of the curriculum development process, with most of the work framed around the formal approval and implementation process required by our institution. New certificate program proposals undergo a multi-step internal review process using a proposal template that includes many of the components addressed above, in addition to a financial plan to support the program. We determined minimum admission criteria: must be an undergraduate enrolled at UC, minimum 2.5 grade point average, must include a personal statement describing educational interests and career goals, and must include one letter of support from a faculty member or professional manager. We identified a program director and a program coordinator to manage administrative tasks and advise students, as well as core faculty for the new courses, supporting their development of syllabi. We collaborated with the Office of Undergraduate Education within the College of Medicine to determine what baseline administrative support they offer, such as course ordering and course evaluation. We also developed an evaluation plan to supplement basic course evaluations; it includes an annual student focus group and alumni survey to evaluate learner satisfaction and career outcomes, identifying areas of strength and improvement.

## 6 Discussion, acknowledgement of limitations, and lessons learned

The six-step curriculum development process achieved several critical goals as we sought to establish an undergraduate training program in CTS. The results of our needs assessment not only supported anecdotal evidence we had from informal exchanges with principal investigators and clinical research directors, but also uncovered important new information we had not previously considered, particularly related to the national landscape (e.g., typical minimum educational requirements for an entry-level job and income trends). Steps one to five allowed us to engage with key stakeholders in meaningful ways, guiding information-gathering and decision-making in order to design the best possible program using national competencies while also leveraging institutional strengths and addressing the unique needs of our local student population.

This project had several limitations. We have not completed the final two steps of the process: implementation and evaluation. The major work of developing the curriculum is accomplished, however, and we anticipate our program proposal will be approved in 2024, with a soft launch of the new program shortly after. A second limitation is the informal way in which we collected qualitative data from stakeholders in needs assessment. We kept meeting notes in this early phase, not recording or transcribing what was said in order to allow for more careful review. We mitigated this limitation by continuing to engage stakeholders throughout, sharing results and obtaining their feedback as the curriculum came together. Future work following the process outlined above would benefit from formalized data collection and analysis procedures so that they may be reported more fully and replicated when desired.

Our CRP Task Force learned several lessons during the development process, which we wish to present here for others working in CTS education. The first is the importance of identifying key stakeholders and working to build bridges and relationships early in the process. Continuous engagement with a diverse group of stakeholders—spanning content experts working in the CTS field, educators knowledgeable about institutional policies and undergraduate student needs, and curriculum development and evaluation experts familiar with CTS workforce development—benefited us throughout the process, helping to identify the right individuals with whom to confer so that we avoided potential missteps. A second lesson learned is to leverage existing resources wherever possible, particularly at large academic institutions where infrastructure to support new training and programming is already in place. Our capacity may have been limited by the grant funding we rely upon to do our work but combining our grant-funded efforts with the vast resources available at our institution expanded our capability far beyond the grant-specific portion. A final suggestion to others pursuing this type of project is not to become too attached to particular plans or ideas early in the process. In our experience, plans shifted regularly throughout each step and an unwillingness to change course or adapt to new information would have hindered progress.

The curriculum development process described by Schneiderhan, Guetterman and Dobson (2019) and followed in the study presented proved an effective framework to develop a new undergraduate certificate program in CTS. It provided a systematic approach to a potentially daunting task, breaking it down into manageable components. Following the process allowed for regular stakeholder engagement and provided a clear path to completion, generating enthusiasm for the positive impact this undergraduate certificate may have on our local CTS workforce.

## Data Availability

The original contributions presented in the study are included in the article/Supplementary material, further inquiries can be directed to the corresponding author.
